# Realistic Vue: a new three-dimensional surface rendering approach for the *in utero* visualization of embryos and fetuses

**DOI:** 10.1590/0100-3984.2018.0050

**Published:** 2019

**Authors:** Eduardo Felix Martins Santana, Edward Araujo Júnior

**Affiliations:** 1 Department of Obstetrics, Escola Paulista de Medicina da Universidade Federal de São Paulo (EPM-Unifesp), São Paulo, SP, Brazil.; 2 Department of Perinatology, Hospital Israelita Albert Einstein, São Paulo, SP, Brazil.

## INTRODUCTION

Three-dimensional (3D) ultrasound has been widely used over the years, because the images obtained with two-dimensional scanning are often not detailed enough to perform highly complex diagnoses^(^^1^^-^^3^^)^. Realistic Vue is a new software application that shows fetal anatomy in high-resolution 3D images with exceptional detail and realistic depth. By controlling luminosity and shading, Realistic Vue makes the intrauterine environment appear more real. In fact, there are growing numbers of studies that show the effective application of realistic 3D ultrasound tools^(^^4^^,^^5^^)^. Early fetal development, morphology, abnormalities, and even behavior can be clearly observed with these new techniques^(^^6^^,^^7^^)^.

We performed a prospective cross-sectional study involving nine singleton embryos/fetuses between 6 and 36 weeks of gestational age. We evaluated the surface rendering provided by Realistic Vue in a high-resolution ultrasound system (WS80; Samsung Medison Co. Ltd., Seoul, South Korea). All ultrasound scans were performed by the same experienced examiner. Only one 3D volume dataset was collected for each pregnant woman. The mean time required to obtain each 3D volume dataset was 90 s, and all rendering images were reconstructed offline. To obtain surface reconstructions of the highest quality, the brightness was adjusted and the appropriate positioning of the virtual light source was verified.

**Figure 1:**
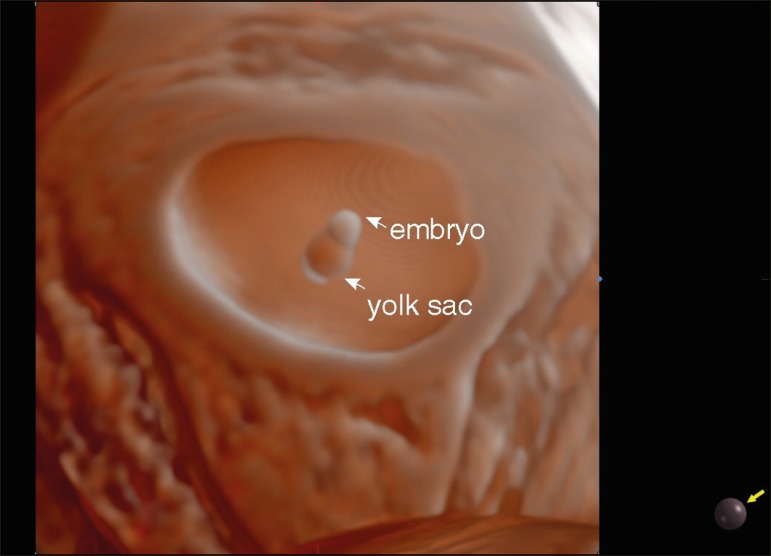
3D Realistic Vue rendering mode image of an embryo and yolk sac at 6 weeks of gestation using transvaginal ultrasonography. Virtual light source position, 2 o'clock. Rotation, 270°.

**Figure 2:**
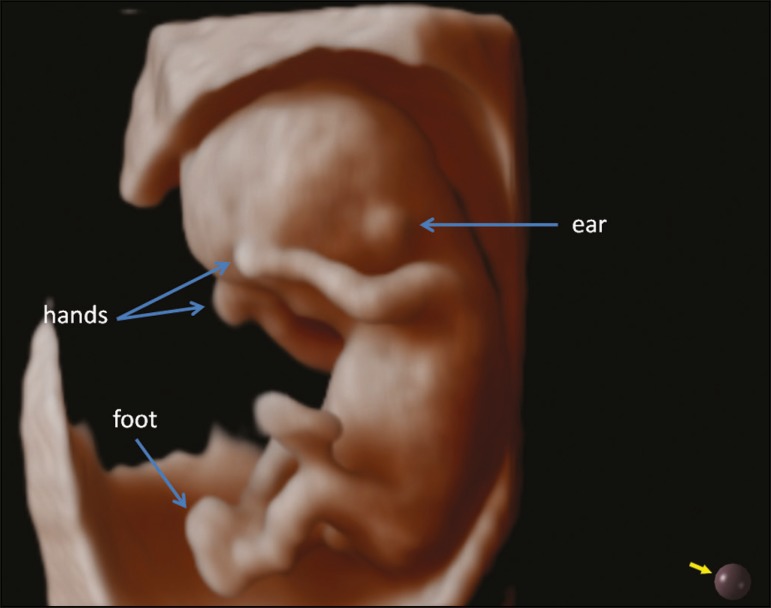
3D Realistic Vue rendering mode image of a fetus at 12 weeks of gestation, showing the hands, feet, and ears. Virtual light source position, 10 o'clock. Rotation, 270°.

**Figure 3:**
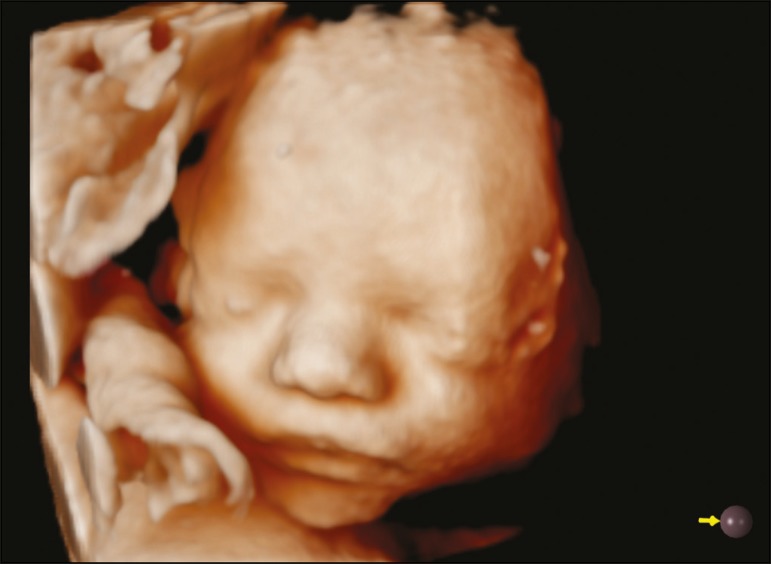
3D Realistic Vue rendering mode image of the face of a fetus at 30 weeks of gestation, showing the eyes, nose and mouth. Virtual light source position, 9 o'clock. Rotation, 270°.

**Figure 4:**
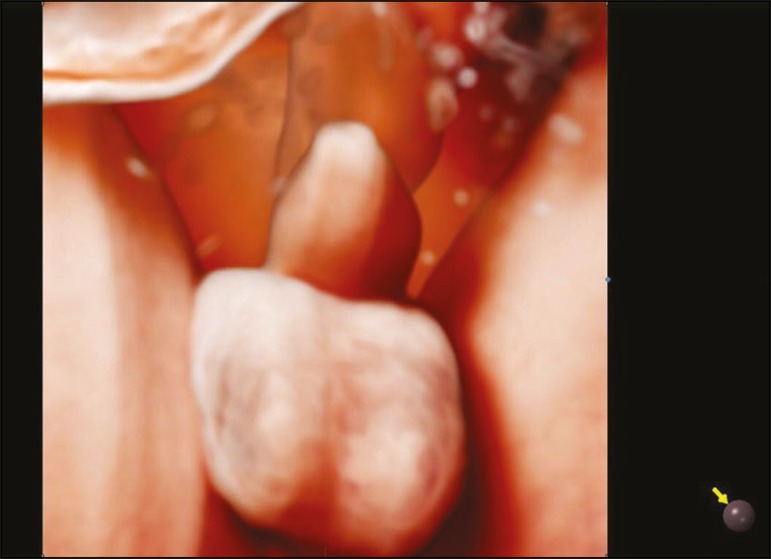
3D Realistic Vue rendering mode image of the genitalia of a male fetus at 30 weeks of gestation, showing the penis and scrotum. Virtual light source position, 11 o'clock. Rotation, 270°.

Realistic Vue allowed realistic imaging of the gestational sac, embryo, and yolk sac at 6 weeks of gestation (Figure 1). At 11 weeks, it was possible to obtain a clear view of the implanted ears, the hands, and the position of the feet (Figure 2). In the third trimester, it was possible to obtain realistic images of the face-eyes, nose, and mouth (Figure 3)-and genitalia-scrotum and penis (Figure 4).

In a previous pictorial essay, Araujo Júnior et al.^(^^5^^)^ described normal embryos/fetuses using the HDlive software (GE Healthcare, Zipf, Austria) with demonstrated realistic surface rendering images similar to those obtained with the Realistic Vue application in the present study. According to Hata et al.^(^^6^^)^, HDlive allows the embryonic development, as well as the fetal facial expressions in the third trimester, to be demonstrated. In fetal congenital anomalies, HDlive rendering images facilitate parental counseling and improve perinatal management by a multidisciplinary team^(^^8^^)^. The recently developed HDlive Flow with HDlive silhouette mode (GE Healthcare) allows the blood flow within a fetal structure to be identified, because of its ability to delineate the blood vessel walls, while the vascular lumina remain transparent^(^^9^^)^. Another recently developed rendering mode, designated Crystal Vue (Samsung Medison Co. Ltd.), is based on image-contrast enhancement that can be used for post-processing of 3D volume datasets, allowing the boundaries between soft tissue and anatomical structures to be delineated^(^^10^^)^. To our knowledge, ours is the first study to use the Realistic Vue application to create realistic surface images of normal embryos/fetuses.

In summary, Realistic Vue allowed realistic images of the embryo/fetus surface between 6 and 36 weeks of gestation. Further studies are needed in order to test the real applications of this software in fetal congenital anomalies or maternal-fetal attachment.
